# Non-thermal atmospheric pressure plasma activates lactate in Ringer’s solution for anti-tumor effects

**DOI:** 10.1038/srep36282

**Published:** 2016-11-08

**Authors:** Hiromasa Tanaka, Kae Nakamura, Masaaki Mizuno, Kenji Ishikawa, Keigo Takeda, Hiroaki Kajiyama, Fumi Utsumi, Fumitaka Kikkawa, Masaru Hori

**Affiliations:** 1 Institute of Innovation for Future Society, Nagoya University, Furo-cho, Chikusa-ku, Nagoya 4648603, Japan; 2 Center for Advanced Medicine and Clinical Research, Nagoya University Hospital, Tsurumai-cho 65, Showa-ku, Nagoya 466-8550, Japan; 3 Department of Obstetrics and Gynecology, Nagoya University Graduate School of Medicine, Tsurumai-cho 65, Showa-ku, Nagoya 466-8550, Japan

## Abstract

Non-thermal atmospheric pressure plasma is a novel approach for wound healing, blood coagulation, and cancer therapy. A recent discovery in the field of plasma medicine is that non-thermal atmospheric pressure plasma not only directly but also indirectly affects cells via plasma-treated liquids. This discovery has led to the use of non-thermal atmospheric pressure plasma as a novel chemotherapy. We refer to these plasma-treated liquids as plasma-activated liquids. We chose Ringer’s solutions to produce plasma-activated liquids for clinical applications. *In vitro* and *in vivo* experiments demonstrated that plasma-activated Ringer’s lactate solution has anti-tumor effects, but of the four components in Ringer’s lactate solution, only lactate exhibited anti-tumor effects through activation by non-thermal plasma. Nuclear magnetic resonance analyses indicate that plasma irradiation generates acetyl and pyruvic acid-like groups in Ringer’s lactate solution. Overall, these results suggest that plasma-activated Ringer’s lactate solution is promising for chemotherapy.

Plasma, the fourth fundamental state of matter, is the most common form of matter in the universe and is visible in such natural phenomena as lightning and the aurora. Non-thermal plasma, such as that found in fluorescent lamps, is generated at low pressure because, in order for the gases to be ionized, the density of particles must be low when energy is added to matter^1^,^2^,^3^. In contrast, plasma like that used in arc welding is generated at high temperature under atmospheric pressure^4^,^5^,^6^. However, an innovative approach using non-thermal atmospheric pressure plasma has been developed and used in industrial applications, such as the micro-fabrication of electronic substrates^7^,^8^,^9^. Plasma medicine is a new field that uses non-thermal atmospheric pressure plasma for a variety of medical applications^10^,^11^,^12^,^13^,^14^,^15^,^16^,^17^,^18^,^19^, such as sterilization^20^,^21^,^22^,^23^,^24^, wound healing^25^,^26^,^27^,^28^,^29^, blood coagulation^30^,^31^,^32^, and cancer treatment^33^,^34^,^35^,^36^,^37^,^38^,^39^.

We previously developed non-thermal atmospheric pressure plasma for medical applications^35^,^40^. Cold plasma affects biological targets not only directly but also indirectly through the medium, which has broadened the potential applications of non-thermal atmospheric pressure plasma in medicine^41^,^42^,^43^,^44^,^45^,^46^,^47^,^48^,^49^,^50^. We have demonstrated that plasma-irradiated medium, also called plasma-activated medium (PAM), has anti-tumor effects against glioblastoma^51^,^52^, as well as ovarian^53^,^54^, gastric^55^, pancreatic^56^, and lung cancer cells^57^. Plasma interacts with oxygen, nitrogen, and water in air to produce various radicals, such as hydroxyl radicals and nitric oxide. In the liquid phase, reactive species with a relatively long lifetime, such as hydrogen peroxide, nitrites, and nitrates, are produced by plasma–liquid interactions^41^,^58^,^59^. In addition to these species that are generally produced by plasma irradiation of liquid, specific species that vary depending on the solution are important for anti-tumor effects. Plasma-activated solutions with simple compositions, such as phosphate-buffered saline (PBS), might be better for treating cancer than more complex solutions^47^. However, the exact species responsible for the anti-tumor effects remain unclear.

In this study, we created plasma-activated Ringer’s lactate solution and investigated its anti-tumor effects on cancer cells. Ringer’s lactate solution has only four components in addition to water: lactate, NaCl, KCl, and CaCl_2_. Systematic analyses demonstrated that lactate is the only anti-tumor component in Ringer’s lactate solution that is activated by plasma, and that plasma irradiation generates acetyl and pyruvic acid-like groups in the solution. For clinical applications, the components of solutions should be as simple as possible, and the species that are responsible for anti-tumor effects should be known. These results represent significant progress toward the use of plasma-activated liquids for clinical applications.

## Results

### Plasma-activated Ringer’s lactate solution has anti-tumor effects

**Figure 1:**
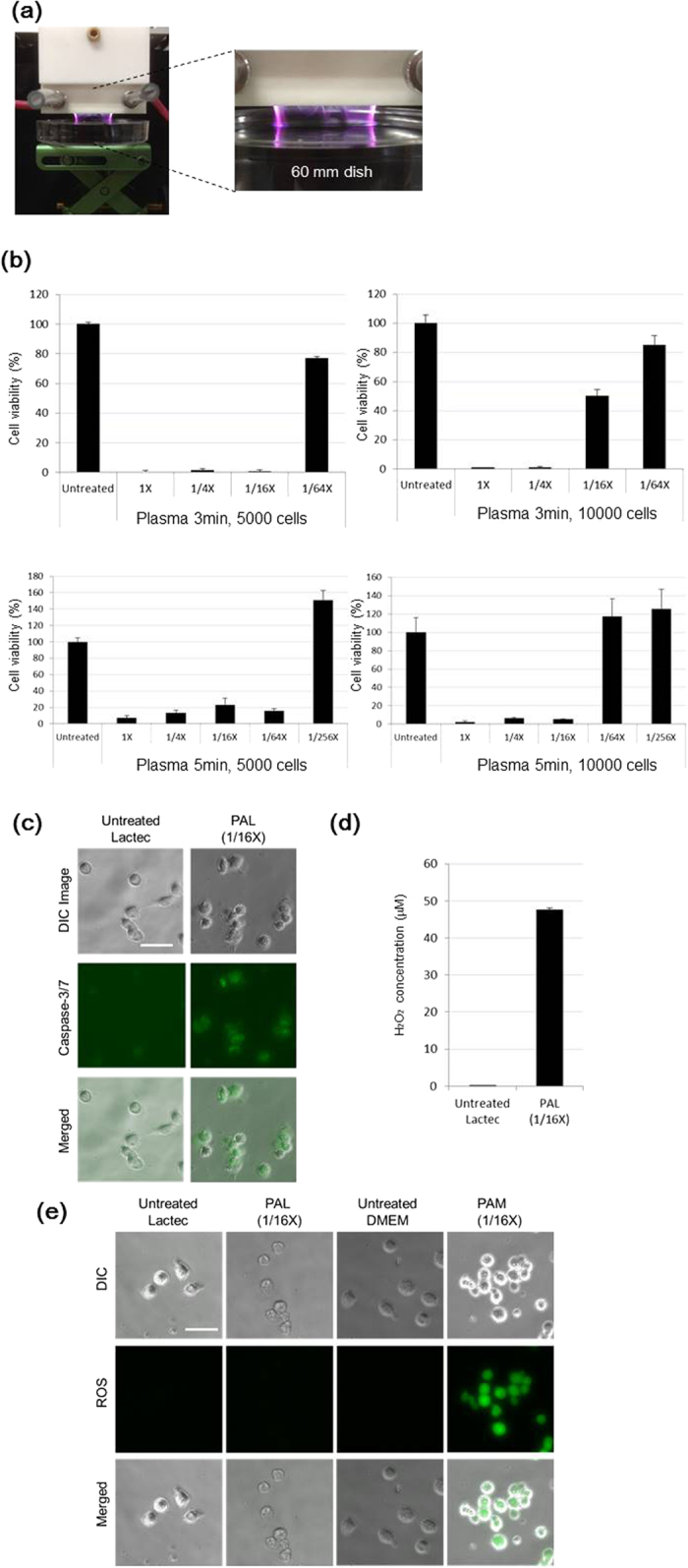
Plasma-activated Ringer’s lactate solution exhibited anti-tumor effects on glioblastoma cells. (**a**) 8 mL Ringer’s lactate solution (lactec) in a 60 mm dish was treated with plasma for 3 or 5 min, with a distance of 3 mm between the plasma source and the surface of the liquid. (**b**) 5000 or 10000 U251SP cells were seeded in a 96-well plate. On the following day, 8 mL lactec in a 60 mm dish was treated with plasma (L = 3 mm, 2.0 slm), and the PAL was diluted 4, 16 or 64 times with lactec. The medium of the cells was replaced with 200 μL of these PALs. After 2 h, PALs was replaced with 200 μL of culture medium. On the following day, cell viability was assayed. (**c**) 10000 U251SP cells were seeded in an 8-well chamber slide. On the following day, 8 mL lactec in a 60 mm dish was treated with plasma (L = 3 mm, 2.0 slm), and the PAL and untreated lactec were diluted 16 times with lactec. The medium of the cells was replaced with 200 μL of the PAL and untreated lactec. After 2 h, the PAL and untreated lactec were replaced with 200 μL culture medium, Cell Event Caspase 3/7 detection reagent was added, and the cells were incubated for 2 h at 37 °C. The cells were observed. (**d**) The H_2_O_2_ concentration of PAL (generated by irradiation for 5 min) diluted 16 times was measured. Data are mean ± SEM. (**e**) 10000 U251SP cells were seeded in an 8-well chamber slide. On the following day, the medium of the cells was replaced with 200 μL of CM-H_2_DCFDA (10 μM) in PBS. Lactec (8 mL) in a 60 mm dish was treated with plasma (L = 3 mm, 2.0 slm), and the PAL was diluted 16 times with lactec. After 1 h, the 200 μL CM-H_2_DCFDA (10 μM) in PBS was replaced with the PAL and untreated lactec. After 2 h, the PALs or untreated lactec were replaced with 200 μL culture medium. After 2 h, the cells were observed.

We previously created PAM to induce apoptosis in cancer cells^51^. However, cell culture medium contains many components, and the reaction products in PAM are complicated and unsuitable for clinical applications. To simplify the components of PAM, we treated Ringer’s lactate solution (Lactec), which has only four components in addition to water, with plasma (Fig. 1a). We called this solution plasma-activated lactec (PAL). When Ringer’s lactate solution was treated with plasma for 3 min, a 16-fold dilution of PAL killed 5000 U251SP glioblastoma cells, and a 64-fold dilution of PAL killed ~20% of 5000 U251SP cells. A four-fold dilution of PAL killed 10000 U251SP cells, and a 16-fold dilution of PAL killed ca. 50% of 10000 U251SP cells (Fig. 1b). When Ringer’s lactate solution was treated with plasma for 5 min, a 64-fold dilution of PAL killed 5000 U251SP glioblastoma cells, but a 256-fold dilution of PAL did not kill any cells. A 16-fold dilution of PAL killed 10000 U251SP cells, but a 64-fold dilution of PAL did not kill any U251SP cells (Fig. 1b). These results suggest that PAL has anti-tumor effects on glioblastoma cells.

We were interested in the mechanisms of cell death caused by PAL. We detected cleaved Caspase3/7 in PAL-treated glioblastoma cells (Fig. 1c), suggesting that PAL induced apoptosis. H_2_O_2_ is generally a major anti-tumor factor in plasma-activated solutions. We measured the concentration of H_2_O_2_ in PAL, and found that PAL diluted 16 times contained 50 μM H_2_O_2_ (Fig. 1d), which is sufficient to kill glioblastoma cells. However, interestingly, we detected little intracellular ROS in PAL-treated glioblastoma cells, while plasma-activated medium (PAM) diluted 16 times induced more ROS than did PAL diluted 16 times (Fig. 1e).

The duration of a solution's anti-tumor effectiveness is important for clinical applications, and we previously found that the duration of the effectiveness of PAM is more than 8 h but less than 18 h^51^. However, if PAM is frozen at less than −80 °C, PAM remains effective for more than 3 months (unpublished data). We are interested in the effectiveness of plasma-activated solution after freeze-thaw cycles for clinical applications. Ringer’s lactate solution treated with plasma for 5 min was subsequently frozen (at −150 °C) and thawed twice. The viability of cells treated with the frozen–thawed solutions was then measured using the MTS assay and compared with that of cells treated with fresh PAL (see Supplementary Fig. 1). For both fresh and frozen/thawed PALs, a 16-fold dilution of PAL killed 10000 U251SP cells, but a 64-fold dilution of PAL did not kill any U251SP cells. These results suggest that PAL is effective after at least two freeze–thaw cycles, and that frozen PAL can be used for clinical applications.

### Lactate is the only anti-tumor component in Ringer’s lactate solution

**Figure 2:**
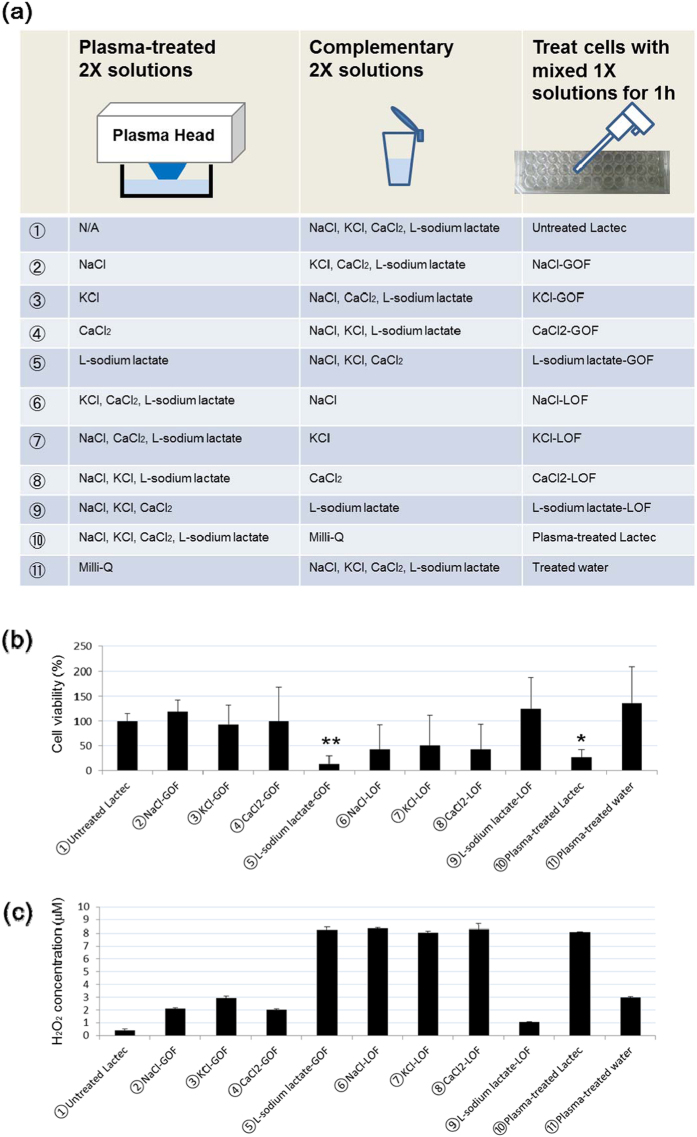
Identification of components in PAL having anti-tumor effects. (**a**) Schematic of experiments for identifying the anti-tumor components in Ringer’s lactate solution by plasma irradiation. Each doubly concentrated NaCl, KCl, CaCl_2_ and L-sodium lactate solution was treated with plasma for 2 min and then mixed with the complementary doubly concentrated solutions. These solutions are referred to as NaCl-GOF, KCl-GOF, CaCl_2_-GOF and L-sodium lactate-GOF (②–⑤). Each doubly concentrated solution lacking NaCl (KCl+CaCl_2_+L-sodium lactate), KCl (NaCl+CaCl_2_+L-sodium lactate), CaCl_2_ (NaCl+ KCl +L-sodium lactate) or L-sodium lactate (NaCl+ KCl + CaCl_2_) was treated with plasma for 2 min, and mixed with the complementary doubly concentrated solutions. These solutions are referred to as NaCl-LOF, KCl-LOF, CaCl_2_-LOF and L-sodium lactate-LOF (⑥–⑨). Doubly concentrated Ringer’s lactate solution was treated with plasma and mixed with the same volume of Milli-Q water (⑩), and vice versa (⑪). (**b**) 10000 U251SP cells were seeded in 200 μL medium in a 96-well plate. On the following day, the medium of the cells in the 96-well plate was replaced with 200 μL of the solutions described in (a). After 1 h, these solutions were replaced with 200 μL culture medium. On the following day, cell viability was measured by the MTS assay and calculated as a percentage of surviving cells relative to control. Data are mean ± SEM. *P, 0.05, **P, 0.01 versus control. (**c**) The H_2_O_2_ concentrations of these solutions were measured. Data are mean ± SEM.

Ringer’s lactate solution contains NaCl (6.0 g/L), KCl (0.3 g/L), CaCl_2_ (0.2 g/L), and L-sodium lactate (3.1 g/L). To identify the anti-tumor components, we systematically produced synthetic plasma-activated solutions (Fig. 2a). Water is the most important component exhibiting anti-tumor effects in plasma-activated medium or solutions, so we carefully chose conditions in which plasma-activated water does not exhibit anti-tumor effects. Each doubly concentrated NaCl, KCl, CaCl_2_, and L-sodium lactate solution was treated with plasma for 2 min, and mixed with the complementary doubly concentrated solutions. These solutions are referred to as gain of function (GOF) solutions: NaCl-GOF, KCl-GOF, CaCl_2_-GOF, and L-sodium lactate-GOF (②–⑤). Each doubly concentrated solution lacking NaCl (KCl+CaCl_2_+L-sodium lactate), KCl (NaCl+CaCl_2_+L-sodium lactate), CaCl_2_ (NaCl+ KCl +L-sodium lactate) or L-sodium lactate (NaCl+ KCl + CaCl_2_) was treated with plasma for 2 min, and mixed with the complementary doubly concentrated solutions. These solutions are referred to as loss of function (LOF) solutions: NaCl-LOF, KCl-LOF, CaCl_2_-LOF, and L-sodium lactate-LOF (⑥–⑨). Doubly concentrated Ringer’s lactate solution was treated with plasma and mixed with the same volume of Milli-Q water (⑩), and vice versa (⑪). Of the GOF solutions, only L-sodium lactate-GOF exhibited anti-tumor effects on U251SP cells. L-sodium lactate-LOF had no anti-tumor effects on U251SP cells (Fig. 2b). The mixture of doubly concentrated plasma-treated water and doubly concentrated Ringer’s lactate solution did not exhibit any anti-tumor effects. These results suggest that L-sodium lactate is activated by plasma and that the activated form has anti-tumor effects on U251SP cells.

H_2_O_2_ is an anti-tumor factor that is typically generated in solutions by plasma treatment^47^,^57^,^60^. To investigate whether H_2_O_2_ generated in PAL is responsible for the observed anti-tumor effects, we measured the H_2_O_2_ concentrations of these solutions (Fig. 2c). Interestingly, L-sodium lactate-GOF contained a higher concentration of H_2_O_2_ (~8 μM) than plasma-treated water, NaCl-GOF, KCl-GOF or CaCl_2_-GOF, while L-sodium lactate-LOF contained a lower concentration of H_2_O_2_ (~1 μM) than plasma-treated Ringer’s lactate solution, NaCl-LOF, KCl-LOF or CaCl_2_-LOF. These results suggest that L-sodium lactate contributes to the generation of H_2_O_2_ by plasma treatment. However, according to our previous results, a H_2_O_2_ concentration above 30 μM is needed to kill 10000 U251SP cells^60^. These results suggest that other components generated by plasma treatment are also responsible for the anti-tumor effects of PAL.

### NMR analyses reveal that plasma irradiation generates acetyl and pyruvic acid-like groups

**Figure 3:**
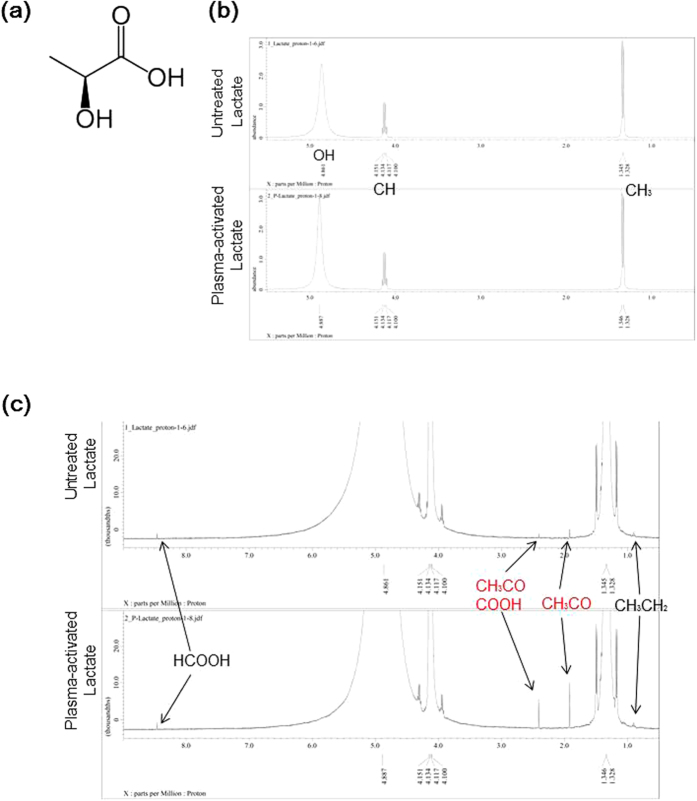
NMR analyses of plasma-activated lactate. (**a**) Chemical structure of lactate. (**b**) ^1^H NMR spectra of untreated lactate and plasma-activated L-sodium lactate. L-sodium lactate (8 mL) in a 60 mm dish was treated with plasma (L = 3 mm, 2.0 slm) for 5 min. The ^1^H NMR and ^13^C NMR spectra of 0.6 mL L-sodium lactate and plasma-activated L-sodium lactate were measured. (**c**) Magnified ^1^H NMR spectra of untreated lactate and plasma-activated L-sodium lactate.

To investigate what products are specifically generated in plasma-activated Ringer’s lactate solution, the ^1^H-NMR spectra of untreated and plasma-treated L-sodium lactate were compared. L-sodium lactate (8 mL) in a 60 mm dish was treated with plasma (the distance between the plasma source and the samples: L = 3 mm, 2.0 standard liters/min (slm)) for 5 min. L-sodium lactate contains OH, CH and CH_3_ groups (Fig. 3a). These groups were detected in both the untreated and plasma-activated L-sodium lactate (Fig. 3b), suggesting that most of the plasma-treated L-sodium lactate was not activated by plasma treatment. However, plasma-activated L-sodium contained more acetyl (CH_3_CO) and pyruvic acid-like groups (CH_3_COCOOH) (Fig. 3c). These results suggest that the acetyl and pyruvic acid-like groups are potential candidates for the anti-tumor factors generated by plasma treatment.

### Various cell lines display different sensitivities to plasma-activated Ringer’s lactate solution

**Figure 4:**
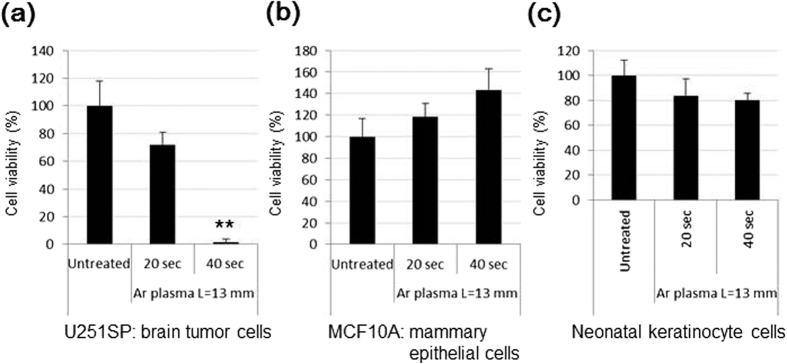
Effects of PAL on various cell lines. 10000 U251SP glioblastoma cells (**a**), MCF10A mammary epithelial cells (**b**), and neonatal keratinocyte cells (**c**) were seeded in a 96-well plate. On the following day, Ringer’s lactate solution (3 mL) in a 6-well plate was treated with plasma for 20 or 40 s using a distance of 13 mm between the plasma source and the surface of the liquid. The medium of the cells in the 96-well plate was replaced with 200 μL of these PALs. As the control, the medium was replaced with 200 μL of untreated Ringer’s lactate solution. After 2 h, PALs or untreated Ringer’s lactate solution was replaced with 200 μL of culture medium. On the following day, cell viability was assayed.

Selective killing of cancer cells is a desirable form of cancer therapy. To investigate the potential of plasma-activated Ringer’s lactate solution, we treated MCF10A mammary epithelial cells and neonatal keratinocyte cells with PAL (Fig. 4). When Ringer’s lactate solution was treated with plasma for 40 s, the PAL effectively killed U251SP cells, but did not affect the epithelial and keratinocyte cells. These results suggest that various cell lines display different sensitivities to PAL.

### Plasma-activated Ringer’s lactate solution exhibits anti-tumor effects *in vivo*

**Figure 5:**
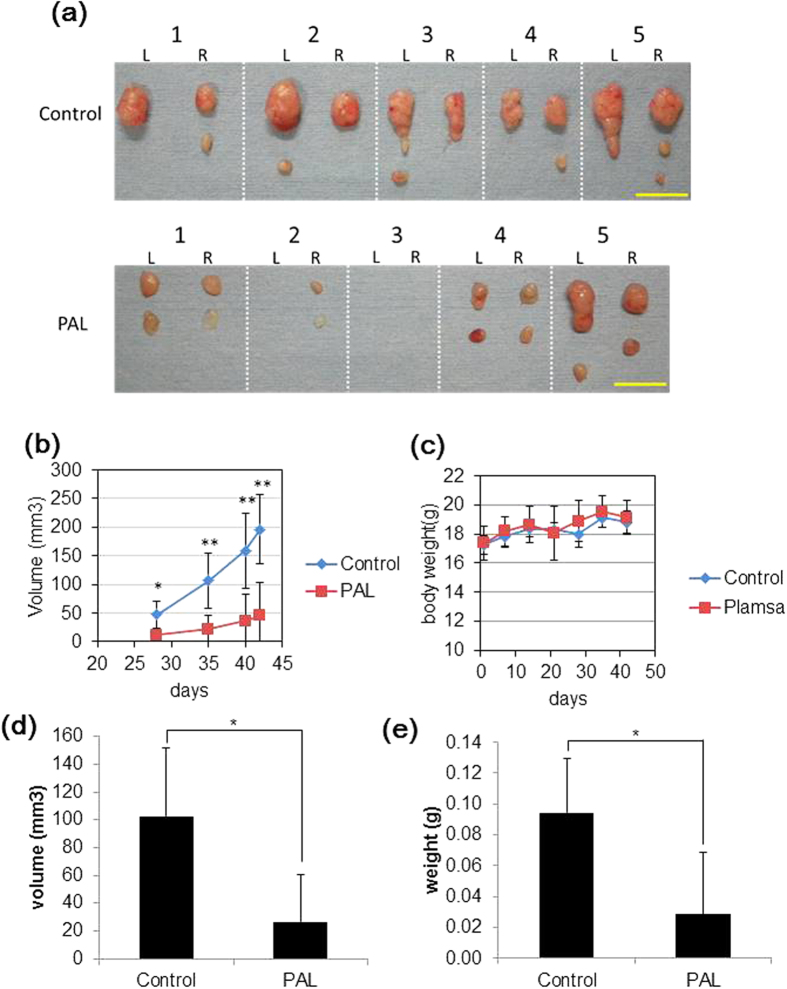
Anti-tumor effects of PAL in mice injected with SiHa cells (human cervical cancer cell line). Female Balb/c nude mice inoculated with 1.5 × 10^3^ SiHa cells with Matrigel in both hind flanks were administered 200 μL of PAL, or Lactec as a control, three times weekly for six weeks. (**a**) Representative images of tumors at the study endpoint, day 42. L and R indicate the location of the tumor (left- or right-side hind flank, respectively). Bars = 1 cm. (**b**) Starting at day 28 after inoculation, tumor size was calculated once a week. (**c**) Body weight was measured once a week during the experiment. (**d**) Tumors were measured and weighed at the endpoint of the study. Data are mean ± SD. *P, 0.05, **P, 0.01 versus control.

To investigate the effectiveness of PAL *in vivo*, we created a mouse xenograft model in which SiHa cells were injected into mice subcutaneously, and the resultant tumors were treated with PAL three times a week for 6 weeks (see Supplementary Fig. 2). PAL effectively reduced tumor volumes (Fig. 5a,b,d,e), and the weights of PAL-treated mice were nearly the same as those of control mice (Ringer’s lactate solution-treated mice). No apparent adverse effects were observed in the mice, which indicates that PAL is safe and effective.

### Plasma-activated acetic acid Ringer’s solution exhibits anti-tumor effects

**Figure 6:**
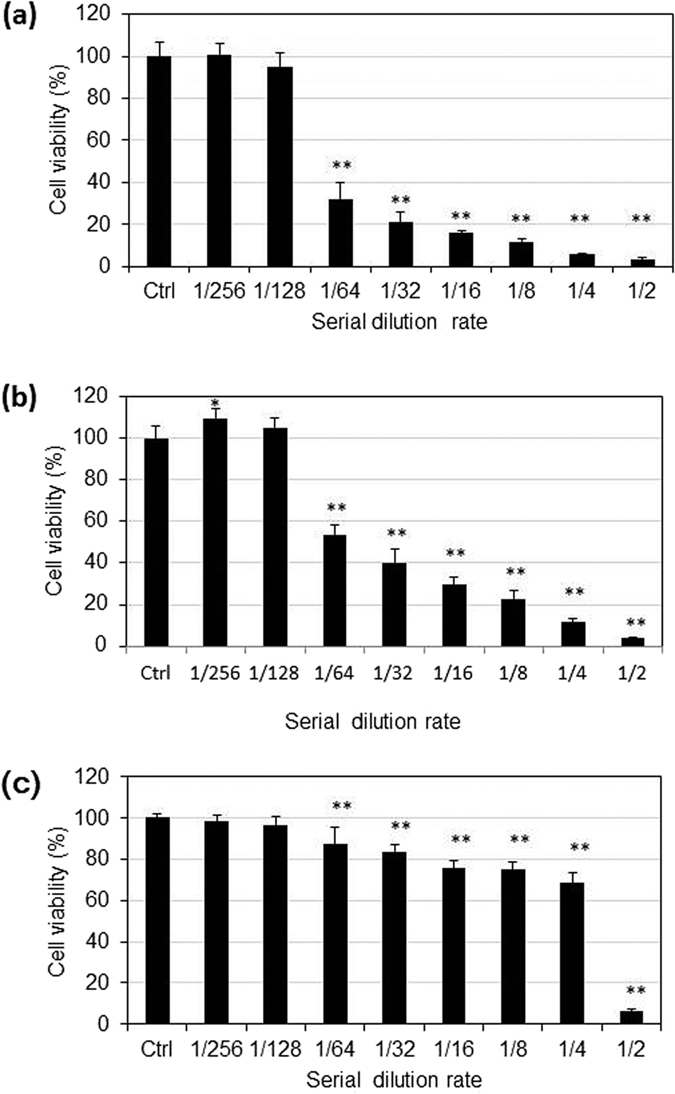
Anti-tumor effects of various plasma-activated Ringer’s solutions. SK-OV-3 cells were treated with serially diluted plasma-activated lactate (**a**), acetate (**b**) or bicarbonate (**c**) ringer for 1 h. After treatment, the Ringer's solution was replaced with fresh medium and the cells were incubated for another 24 h. Cell viability was measured by the MTS assay and calculated as a percentage of surviving cells relative to control. Data are mean ± SD. *P, 0.05, **P, 0.01 versus control.

Several Ringer’s solutions have been developed for clinical use. We investigated the anti-tumor effects against the ovarian cancer cell line SK-OV-3 using plasma-activated acetic acid Ringer’s solution (PAA) and plasma-activated bicarbonate Ringer’s solution (PAB) in addition to plasma-activated Ringer’s lactate solution (PAL) (Fig. 6). Interestingly, PAL and PAA effectively killed SK-OV-3 cells, whereas PAB did not. These results suggest that PAL and PAA are important products for obtaining anti-tumor effects.

## Discussion

New cancer therapies are needed to avoid the side effects commonly seen with traditional surgery, chemotherapy, and radiation therapy. One new approach is the use of non-thermal atmospheric pressure plasma. Plasma can affect cells both directly and indirectly. Direct treatment of cancer using plasma is an important approach, and the first clinical application of plasma for cancer treatment has recently been reported^61^. Indirect treatment of cancer using plasma via solutions has been recently recognized as an important new chemotherapy^41^,^42^,^43^,^62^.

We previously proposed plasma-activated medium (PAM) as a new chemotherapy^41^,^42^,^43^,^51^,^53^,^63^. Several studies have demonstrated that plasma-activated solutions generally contain H_2_O_2_ that is generated by the interaction between plasma and water, and H_2_O_2_ is responsible for the anti-tumor effect^50^,^57^,^60^; however, some studies have suggested that components other than H_2_O_2_ in PAM are also responsible for anti-tumor effects. Plasma-activated solutions such as PAM, as well as direct plasma treatment, induce intracellular reactive oxygen species (ROS), and ROS is responsible for the anti-tumor effects^53^,^64^. Interestingly, PAL induced less ROS than did PAM. Even PAL that contains around 50 μM H_2_O_2_ induced little intracellular ROS in glioblastoma cells. PAL that contains around 8 μM H_2_O_2_ exhibited an anti-tumor effect on U251SP glioblastoma cells, whereas at least 30 μM H_2_O_2_ is required to observe anti-tumor effects on U251SP in the absence of plasma treatment^60^. PAL also induced apoptosis, as did PAM. These results show that PAL induced apoptosis without inducing intracellular ROS, suggesting that PAL induced apoptosis through different mechanisms than PAM-induced apoptosis.

As Ringer’s solutions are already used clinically, we developed a new PAM for clinical applications by irradiating Ringer’s lactate solution with plasma. In the present study, we demonstrated that plasma-activated Ringer’s lactate solution has anti-tumor effects *in vitro* and *in vivo*. Moreover, Ringer’s lactate solution is a simple solution containing only four components in addition to water. We found that only the L-sodium lactate irradiated by plasma had anti-tumor effects. This is a reasonable result because NaCl, KCl, and CaCl_2_ are commonly found in other solutions, and they do not show anti-tumor effects following plasma treatment. Interestingly, L-sodium lactate but not NaCl, KCl, and CaCl_2_ in solution contributes to the generation of H_2_O_2_ by plasma irradiation, suggesting that increased H_2_O_2_ through activation of lactate might be partially responsible for the anti-tumor effects by plasma-activated Ringer’s lactate solution.

Plasma treatment of lactate generates acetyl and pyruvic acid-like groups. The present study may be the first demonstration that acetyl and pyruvic acid-like groups generated by plasma treatment exhibit anti-tumor effects. Like PAL, plasma-activated acetic acid Ringer’s solution (PAA) also exhibits anti-tumor effects. Thus, plasma treatment likely produces acetyl and pyruvic acid-like groups in acetic acid Ringer’s solution. These speculations should be tested by NMR analyses of plasma-activated acetic acid in the future. These results suggest that it might be possible to use plasma treatment to design specific chemical compounds with anti-tumor effects.

## Methods

### Cell lines and culture

U251SP cells (human glioblastoma cell line) and MCF10A cells (human mammary epithelial cell line) were grown in Dulbecco's Modified Eagle Medium (Sigma-Aldrich, St. Louis, MO) supplemented with 10% fetal bovine serum (FBS) and penicillin (100 U/mL)-streptomycin (100 μg/mL; P/S). SiHa cells (a human cervical cancer cell line) and SK-OV-3 cells (a human ovarian cancer cell line) were grown in Roswell Park Memorial Institute 1640 (Sigma-Aldrich) supplemented with 10% FBS and P/S). Human neonatal keratinocyte cells (purchased from ATCC, Manassas, VA) were grown using a keratinocyte growth kit (purchased from ATCC, Manassas, VA) under an atmosphere of 5% CO_2_ at 37 °C.

### Preparation of plasma-activated Ringer’s lactate solution

The experimental setup to prepare the plasma-activated Ringer’s lactate solution is shown in Fig. 1a and has been previously described^51^. While argon gas was flowing, plasma in the main discharge region was excited by applying 10 kV from a 60-Hz commercial power supply to two electrodes 20 mm apart. The flow rate of argon gas was set at 2 standard liters/min (slm), and the distance between the plasma source and the samples was fixed at L = 3 or 13 mm.

### Measuring H_2_O_2_ concentrations

H_2_O_2_ concentrations were determined using a calibration curve constructed from known stock H_2_O_2_ solutions (0, 5, 10, and 20 μΜ). The samples were measured in triplicate using an Amplex Red reagent kit (Life Technologies, Carlsbad, CA, USA) and by monitoring the peak fluorescence emission at 560 nm using a microplate reader (POWERSCAN HT, DS Pharma Biomedical, Kirkland, WA).

### Cell viability assay

The effect of PAL on cell viability was measured using an Aqueous One Solution Cell Proliferation Assay kit (Promega, Madison, WI) according to the manufacturer’s instructions. Absorbance was measured at 490 nm with a POWERSCAN HT microplate reader. Cells were seeded in 200 μL medium in a 96-well plate. On the following day, 8 mL Ringer’s lactate solution in a 60 mm dish or 3 mL Ringer’s lactate solution in a 6-well plate was treated with plasma (L = 3 mm or 13 mm, 2.0 slm), and the medium of the cells in the 96-well plate was replaced with 200 μL of these PALs. After 2 h or 1 h, PAL was replaced with 200 μL of the culture medium. On the following day, cell viability was assayed using the cell proliferation assay kit. The absorbance values were averaged over three independent experiments, and data are expressed as the mean ± SEM.

### Detection of apoptosis

Cells were seeded in 200 mL medium in an 8-well culture slide. On the following day, 8 mL Ringer’s lactate solution in a 60 mm dish or 3 mL Ringer’s lactate solution in a 6-well plate was treated with plasma (L = 3 mm or 13 mm, 2.0 slm) and the plasma-activated Ringer’s lactate solutions were diluted 16 times with Ringer’s lactate solution. The medium of the cells in the 96-well plate was replaced with 200 μL of these PALs. After 2 h or 1 h, PAL was replaced with 200 μL of the culture medium, and Cell Event Caspase 3/7 detection reagent (5 μM, Invitrogen, Carlsbad, CA, USA) was added and the cells were incubated for 2 h at 37 °C. The cells were observed using a Keyence BZ9000 microscope (Osaka, Japan).

### Detection of intracellular ROS

Ten thousand U251SP cells were seeded in an 8-well chamber slide in 200 μl of culture medium. On the following day, the medium of the cells in the 8-well chamber slide was replaced with 200 μL of CM-H_2_DCFDA (Life Technologies, Carlsbad, CA, USA) (10 μM) in PBS. Eight milliliters Ringer’s lactate solution in a 60 mm dish was treated with plasma (L = 3 mm, 2.0 slm) and the plasma-activated Ringer’s lactate solution was diluted 16 times with Ringer’s lactate solution. After 1 h, 200 μL of CM-H_2_DCFDA (10 μM) in the cell culture chambers was replaced with this 16 times diluted PAL. As a control, 200 μL of the CM-H_2_DCFDA (10 μM) in the cell culture chambers was replaced with 200 μL of untreated Ringer’s lactate solution. After 2 h, these PALs and untreated Ringer’s lactate solution were replaced with 200 μL culture medium. After 2 h, the cells were observed using the Keyence BZ9000 microscope.

### NMR analyses

L-sodium lactate (8 mL) in a 60 mm dish was treated with plasma (L = 3 mm, 2.0 slm) for 5 min. The ^1^H NMR and ^13^C NMR spectra of 0.6 mL L-sodium lactate and plasma-activated L-sodium lactate were measured using a JNM-ECX 400 (JEOL RESONANCE, Tokyo, Japan). The spectrometer was operated at 399.8 MHz for ^1^H nuclei and 100.5 MHz for ^13^C nuclei.

### Animal studies

Eight-week-old female nude mice (BALB/C) (N = 10) were obtained from Japan SLC (Nagoya, Japan). A total of 1.5 × 10^3^ SiHa cells were suspended in 150 μL of serum-free medium and 150 μL of Matrigel (BD Biosciences, San Jose, CA), and subcutaneously injected into both hind flanks of the mice. The mice were then randomly divided into two equal-sized groups. One group of mice received 200 μL of PAL by subcutaneous injection in each hind flank and the other group received the same volume of non-plasma-irradiated Lactec as a control group. In this animal study, PAL was prepared as follows: 5.5 mL of Lactec was placed in a 21-mm dish and irradiated with plasma for 10 min. Treatment with PAL injections was repeated three times a week starting 24 h after cell injection. To evaluate the anti-tumor effects, the tumor volume was calculated using the formula: π/6 × (largest diameter) × (smallest diameter)^2^. At 42 days after cell injection, the mice were sacrificed and the tumor tissues were harvested and weighed. This animal experiment protocol was approved by the Animal Experimental Committee of the Graduate School of Medicine, Nagoya University (Permission No. 28268). The animal study was carried out in accordance with the Guidelines for Animal Experiments of the Nagoya University School of Medicine. The protocol for these animal experiments was published previously^53^,^56^.

## Additional Information

**How to cite this article**: Tanaka, H. *et al*. Non-thermal atmospheric pressure plasma activates lactate in Ringer's solution for anti-tumor effects. *Sci. Rep.* **6**, 36282; doi: 10.1038/srep36282 (2016).

**Publisher’s note:** Springer Nature remains neutral with regard to jurisdictional claims in published maps and institutional affiliations.

## Supplementary Material

Supplementary Information
