# Supplementary material for: Non-thermal atmospheric pressure plasma activates lactate in Ringer’s solution for anti-tumor effects
Source: Sci Rep. 2016 Nov 8;6:36282. doi: 10.1038/srep36282 (PMC5099972; doi:10.1038/srep36282)
Supplement: Supplementary Information [file srep36282-s1.pdf]

Non-thermal atmospheric pressure plasma activates lactate in Ringer's solution for anti-tumor effects

Hiromasa Tanaka[1, 2, *], Kae Nakamura[3, *], Masaaki Mizuno[2], Kenji Ishikawa[1], Keigo Takeda[1],

Hiroaki Kajiyama[3], Fumi Utsumi[3], Fumitaka Kikkawa[3], and Masaru Hori[1]

*: equally contributed

[1] Institute of Innovation for Future Society, Nagoya University, Furo-cho, Chikusa-ku, Nagoya 464-8603, Japan

[2] Center for Advanced Medicine and Clinical Research, Nagoya University Hospital, Tsurumai-cho 65, Showa-ku, Nagoya 466-8550, Japan

[3] Department of Obstetrics and Gynecology, Nagoya University Graduate School of Medicine, Tsurumai-cho 65, Showa-ku, Nagoya 466-8550, Japan

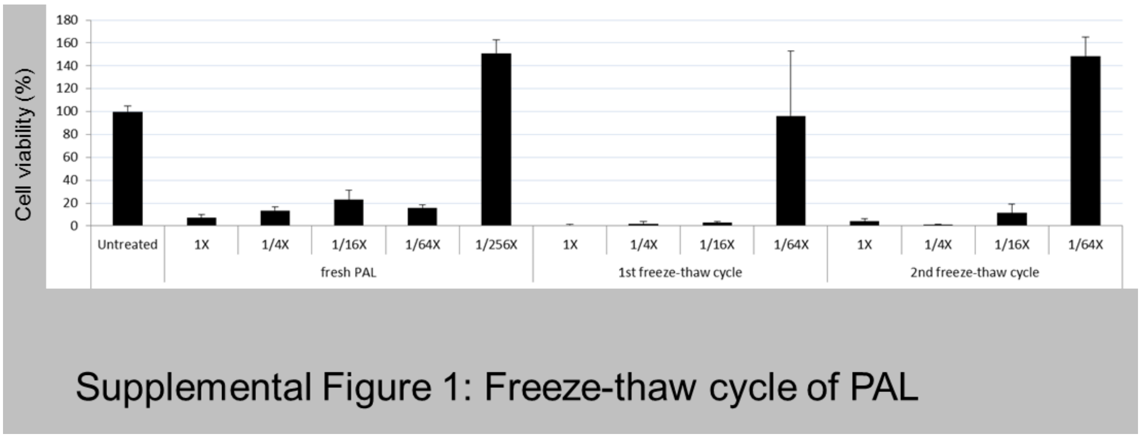

Supplemental Figure 1: Freeze-thaw cycle of PAL

Supplementary Figure 1: Freeze–thaw cycle of PAL.

8 mL Ringer's lactate solution in a 60 mm dish was treated with plasma (L = 3 mm, 2.0 slm) for 5 min, then the PAL was frozen at -150 °C and thawed once (12 days after preparation of the PAL) and then a second time (14 days after preparation of the PAL). The thawed PALs were diluted 4-, 16- or 64-fold with Ringer's lactate. After 2 h, the medium of the cells (5000 U251SP glioblastoma cells seeded the previous day) in a 96-well plate was replaced with 200 μL of these PALs. On the following day, cell viability was measured by the MTS assay and calculated as a percentage of surviving cells relative to control. The absorbance values were averaged over three independent experiments, and data are expressed as the mean ± SEM.

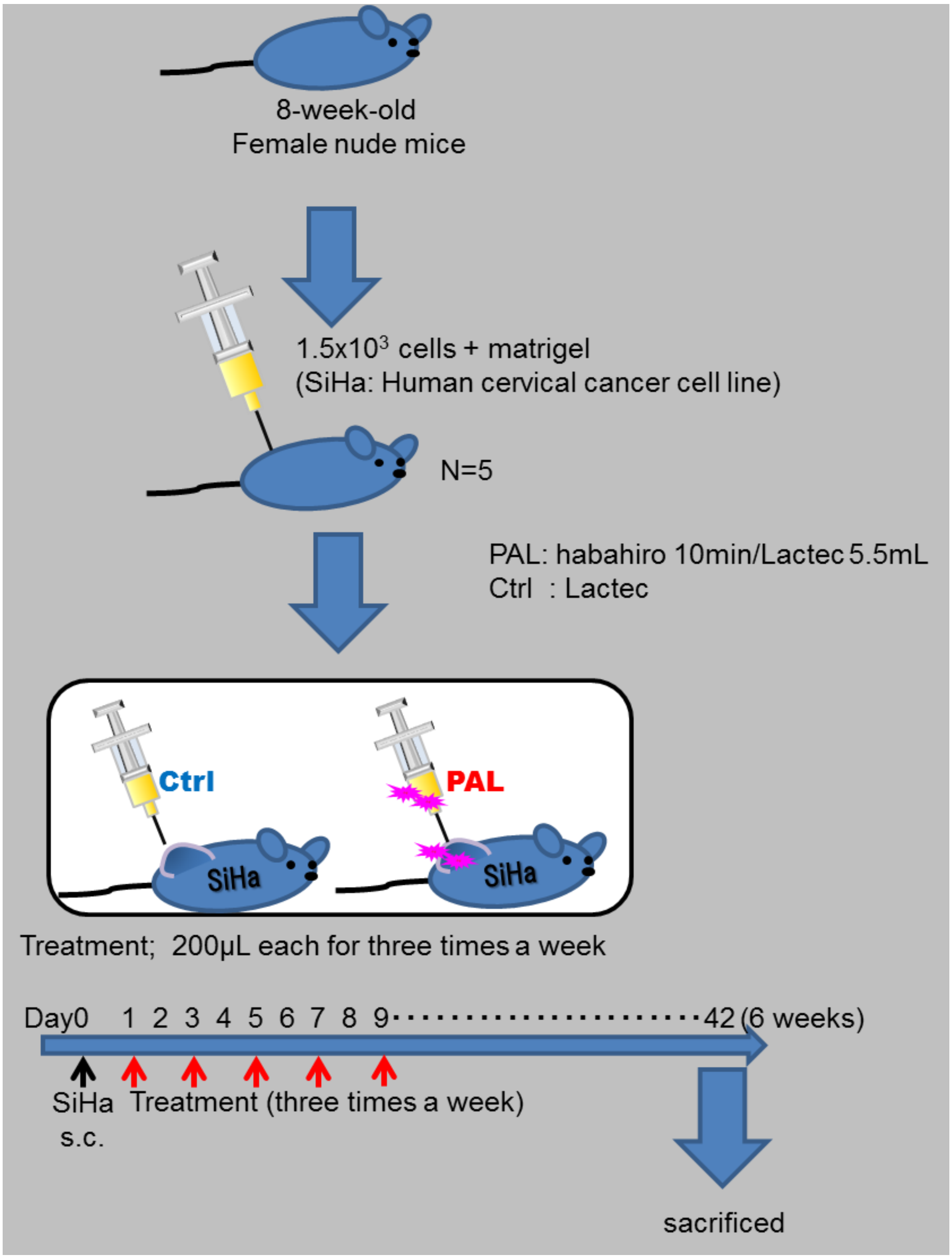

Supplementary Figure 2: PAL treatment plan for mice injected with SiHa cells.

Ten 8-week-old female mice were divided equally into two groups. SiHa cells (1.5×10^3) mixed with Matrigel were injected into both flanks of the mice in each group. Starting the next day, the mice received either 0.2 mL of Ringer's solution alone or plasma-activated Ringer's solution locally

into each hind flank three times a week. All mice were sacrificed at 42 days after injection of the SiHa cells. Red arrowheads on the schedule line indicate treatment points.
